# Peripheral immune profiling identifies developmental immune displacement into an HLA-DR-low monocyte state in necrotizing enterocolitis

**DOI:** 10.3389/fimmu.2026.1868823

**Published:** 2026-07-21

**Authors:** Yihuang Huang, Luyang Hong, Xinhui Guo, Huiyu Chen, Ningxin Luo, Shujuan Li, Junyan Han, Xincheng Cao, Siyuan Jiang, Yun Cao

**Affiliations:** 1 Department of Neonatology, Children’s Hospital of Fudan University, National Children’s Medical Center, Shanghai, China; 2 National Health Commission (NHC) Key Laboratory of Neonatal Diseases, Fudan University, Shanghai, China

**Keywords:** HLA-DR, immune development, mass cytometry, monocytes, necrotizing enterocolitis, preterm infants

## Abstract

**Background:**

Necrotizing enterocolitis (NEC) is a devastating inflammatory disease of prematurity, yet how it reshapes systemic immunity relative to normal postnatal immune maturation remains unclear. We sought to define the peripheral immune architecture of NEC within a developmental framework and to evaluate whether its dominant signal could be linked to intestinal pathology.

**Methods:**

We performed high-dimensional Cytometry by Time-of-Flight profiling on 45 peripheral blood samples from 40 preterm infants spanning birth, postnatal control, pre-onset NEC, NEC onset, and post-onset NEC. Peripheral immune states were modeled relative to a birth-to-postnatal developmental reference trajectory. Key findings were further examined in publicly available intestinal single-cell and bulk transcriptomic datasets, and monocyte human leukocyte antigen-DR (HLA-DR) intensity was assessed as a simplified protein-level metric.

**Results:**

Twenty-seven immune cell subtypes were resolved across major lymphoid and myeloid compartments. In controls, peripheral immunity followed a stereotyped transition from early myeloid predominance toward lymphoid expansion after birth. NEC onset was associated with marked deviation from this trajectory and was dominated by expansion of HLA-DR-low CD16-negative monocytes, contraction of naive and regulatory T-cell subsets, and rewiring of immune correlations toward a myeloid-centered network. Cross-platform analyses supported concordance between the peripheral HLA-DR-low monocyte signal and inflammatory intestinal myeloid states with reduced antigen-presentation programs. Lower monocyte HLA-DR correlated with inflammatory severity; an exploratory within-cohort ROC analysis yielded an area under the curve of 0.84 (95% confidence interval 0.65-0.99).

**Conclusions:**

NEC is associated with developmentally discordant systemic immune remodeling centered on an HLA-DR-low monocyte state in blood, with concordant intestinal myeloid signatures in independent datasets. Monocyte HLA-DR may serve as a mechanistically anchored candidate readout for immune monitoring in preterm infants with NEC, pending external validation.

## Background

Necrotizing enterocolitis (NEC) remains the most devastating gastrointestinal emergency of prematurity, affecting 5-12% of very low birth weight infants and carrying mortality rates of 20-50% (1, 2). Even among survivors, the burden remains substantial, with long-term sequelae including short bowel syndrome, growth failure, and neurodevelopmental impairment. Over the past decade, experimental and translational studies have established that innate immune dysregulation within the immature intestine is central to NEC pathogenesis, particularly through epithelial barrier failure, aberrant microbial sensing, and TLR4-driven inflammatory injury (3, 4). Yet NEC is not only a localized intestinal lesion. In clinical practice, disease progression is accompanied by pronounced systemic inflammatory disturbance, but this host response is typically inferred from bulk laboratory measurements, which provide little insight into the cellular architecture of the circulating immune response. A high-resolution definition of peripheral immune remodeling in NEC therefore remains an important unmet need.

Recent single-cell and high-dimensional studies have begun to illuminate parts of this landscape, but the picture remains incomplete. CyTOF profiling of NEC has highlighted NK-cell activation and systemic immune perturbation (5), whereas intestinal single-cell RNA sequencing has revealed inflammatory macrophage states and extensive epithelial-mesenchymal-immune crosstalk within diseased tissue (6). In parallel, targeted studies have implicated trafficking monocyte populations (7), and recent synthesis work has underscored the broader relevance of the myeloid compartment to NEC biology (8). However, these studies do not yet provide a comprehensive view of the peripheral immune response across the full lymphoid-myeloid spectrum, nor do they establish how circulating immune alterations at NEC onset should be interpreted in the context of immune development in prematurity. As a result, it remains unclear whether the dominant peripheral immune features observed in NEC represent disease-specific remodeling, developmental immaturity, or an interaction between the two.

This distinction is especially important because the neonatal immune system is highly dynamic during the first weeks of postnatal life. In healthy newborns, peripheral immunity follows a reproducible maturational trajectory characterized by progressive remodeling across multiple lineages, with early myeloid predominance gradually giving way to expansion and diversification of lymphoid compartments (9). This transition is shaped by microbial colonization, antigen exposure, and the waning influence of maternally derived immunity. Within this developmental framework, suppressive myeloid populations such as neonatal myeloid-derived suppressor cells help restrain excessive inflammation and maintain immune homeostasis (10). In preterm infants, however, these regulatory programs may be quantitatively or functionally altered under conditions of severe disease. Accordingly, without defining where NEC falls relative to this normal developmental landscape, it is difficult to distinguish disease-specific immune perturbations from changes attributable to developmental timing alone.

Here, we used high-dimensional CyTOF profiling to characterize peripheral blood immunity in preterm infants across five clinically defined states spanning birth, postnatal controls, pre-onset NEC, NEC at onset, and post-onset NEC. We first defined the developmental immune trajectory in non-NEC controls, then quantified how NEC samples deviated from this reference framework at the levels of lineage composition, cell subtype abundance, and inter-cellular coordination. Finally, we integrated these peripheral immune findings with independent intestinal scRNA-seq and bulk RNA-seq datasets to determine whether the dominant peripheral NEC-associated signal mapped onto a corresponding intestinal myeloid program. Through this developmental and cross-compartment framework, we sought to identify the principal immune state associated with NEC onset and to evaluate its relevance for biologic interpretation and future immune monitoring.

## Methods

### Study design, cohort definition, and sample collection

This prospective observational study was conducted in the neonatal intensive care unit (NICU) of Children’s Hospital of Fudan University. Preterm infants born at gestational age <34 weeks and/or with birth weight <1500 g were screened for enrollment. NEC was diagnosed according to modified Bell’s criteria (11), and only infants meeting stage IIA or higher were classified as NEC cases. The study was approved by the institutional ethics committee (Approval No. 2018[149] and 2025[121]), and written informed consent was obtained from parents or legal guardians before sample collection.

Peripheral blood samples (300–500 uL) for the study were obtained from residual clinical blood collected during routine standard-of-care sampling. Exact per-sample blood volumes were not retained, but all valid samples fell within this 300–500 uL range. A total of 57 candidate peripheral blood samples were assessed; 12 were excluded before CyTOF acquisition because of hemolysis (n = 3) or insufficient blood volume (n = 9). The final analytical dataset therefore comprised 45 samples from 40 unique infants. Blood sampling for NEC-at-onset cases coincided with routine clinical evaluation at diagnosis rather than research-only phlebotomy. Because some infants contributed serial samples, group assignment was defined at the sample level, whereas demographic summaries were evaluated at the patient level where appropriate.

Samples were classified into five predefined clinical groups based on disease status and sampling time. In infants who did not develop NEC during the NICU stay, samples collected at day of life (DOL) <=2 were assigned to the Birth group (n = 10), whereas samples collected at DOL >2 were assigned to the postnatal Control group (n = 15). In infants who developed NEC, all samples were obtained after DOL 2 and were categorized as NEC-pre (collected before clinical onset; n = 4), NEC at onset (collected within 48 h of diagnosis; n = 12), or NEC-post (collected after disease onset; n = 4). For within-NEC severity analyses, onset samples were further stratified into NEC stage II subgroup (n = 8) and NEC stage III subgroup (n = 4) according to modified Bell’s criteria (11).

### Sample collection and mass cytometry

Peripheral blood (300–500 uL) was collected into heparin-containing tubes, maintained at 4 °C, and processed for mass cytometry within 30 min to 1 h after blood draw using a standardized surface-staining workflow; exact per-sample volumes were not retained. After antibody staining, cells were labeled with cisplatin (195Pt) for live/dead discrimination, fixed, and labeled with iridium DNA intercalators (191Ir and 193Ir) prior to acquisition. Samples were acquired on a Helios mass cytometer (cytometry by time-of-flight; Standard BioTools, formerly Fluidigm) and recorded as single-cell FCS 3.0 files. This workflow follows common reporting elements for high-dimensional cytometry experiments (minimum information for flow cytometry experiments; MIFlowCyt) adapted to mass cytometry: sample type, antibody panel with reporter isotopes, instrument platform, and documented preprocessing gates.

We designed a 26-marker CyTOF panel to resolve neonatal T, B, NK, and myeloid compartments and selected activation/maturation antigens. CD45 (89Y) was included as a pan-leukocyte marker for leukocyte gating; the phenotyping markers comprised 26 targets. CD193 (175Lu) was included in the initial mass cytometry acquisition for sample-level detection and quality monitoring but was not used in downstream gating, cell-type annotation, or statistical analyses reported in this study. Metal tags are reported as mass channels (isotope reporter). CD193 (175Lu) was included in the initial mass cytometry acquisition for sample-level detection and quality monitoring but was not used in downstream gating, cell-type annotation, or statistical analyses reported in this study:

Lineage markers (15): CD3 (170Er), CD4 (174Yb), CD8 (168Er), CD14 (151Eu), CD16 (165Ho), CD19 (142Nd), CD7 (147Sm), CD11b (209Bi), CD11c (159Tb), CD24 (166Er), CD56 (163Dy), TCRαβ (176Yb), TCRγδ (152Sm), HLA-DR (173Yb), IgA (148Nd).

Differentiation markers (10): CD25 (169Tm), CD27 (150Nd), CD28 (160Gd), CD38 (167Er), CD45RA (155Gd), CD45RO (149Sm), CD127 (143Nd), CD185/CXCR5 (153Eu), IgD (146Nd), IgM (172Yb).

Additional reagents: cisplatin (195Pt) for viability; iridium DNA intercalators (191Ir, 193Ir) for intact single-cell detection.

### Data preprocessing

Events with positive total iridium DNA signal (191Ir + 193Ir) were retained; a threshold was set at the 1st percentile among DNA-positive events. Event length was filtered using robust scatter (median absolute deviation; MAD). Events were retained within median ± 3 × 1.4826 × MAD, with a minimum event length of 10 arbitrary units, to exclude doublets and debris. Live cells were defined by low cisplatin (195Pt) uptake. A high-cisplatin threshold was defined per sample from the distribution of cisplatin-positive events; events below this threshold were classified as viable. CD45+ cells were retained using a per-sample threshold on CD45 (89Y); events below the 5th percentile of CD45 signal were excluded as CD45-negative/debris. For clustering and visualization, protein intensities were transformed with the inverse hyperbolic sine (arcsinh) using cofactor 5 (asinh(x/5)), which stabilizes variance across the dynamic range typical of CyTOF count data.

Per-sample quality-control metrics, including acquired pre-gating event counts, stepwise preprocessing yields, final CD45+ leukocyte counts, and retention rates, are reported in **Supplementary Table 1** and summarized in **Supplementary Figure 1B**. Subtype abundance analyses used both absolute event counts and frequency summaries. For rare subtypes, we prespecified a 20-event per-sample reliability threshold and report the fraction of samples reaching >=10, >=20, and >=50 events for each subtype and group (**Supplementary Table 13**).

### Lineage assignment and subtype annotation

After preprocessing, CD45+ cells were first partitioned into four major immune lineages using a rule-based gating framework applied to arcsinh-transformed marker intensities. T cells were defined as CD3 >2.0 and/or TCRab >2.0, with an additional permissive gate for CD7+CD4+ events to retain low-CD3 T-lineage cells. B cells were defined as CD19 >2.0 in the absence of T-cell and NK-cell markers (CD3 <2.0 and CD56 <2.0). NK cells were defined as CD56 >2.0 with exclusion of T-cell and B-cell markers (CD3 <2.0 and CD19 <2.0). The remaining CD3-CD19-CD56- compartment was assigned to Myeloid + Granulocytes when CD14 >0.5 and/or CD16 >3.0.

To resolve within-lineage heterogeneity, clustering was performed independently for each lineage using FlowSOM (R package FlowSOM v2.4.0). For each lineage, a 10 x 10 self-organizing map (100 nodes) was trained and subsequently aggregated by meta-clustering. The final number of meta-clusters was optimized separately for each lineage to balance resolution and interpretability: T cells, 16; B cells, 8; NK cells, 6; and Myeloid + Granulocytes, 30. A relatively higher resolution was retained for the myeloid/granulocyte compartment to preserve phenotypically distinct HLA-DR-low monocyte states. Lineage-specific clustering features were chosen to emphasize biologically informative variation within each compartment: T cells were clustered using CD3, CD4, CD8, CD45RA, CD45RO, CD27, CD28, CD25, CD127, TCRgd, HLA-DR, CD38, and CD7; B cells using CD19, IgD, IgM, IgA, CD27, CD38, HLA-DR, and CD24; NK cells using CD56, CD16, CD7, CD8, CD45RA, CD38, and HLA-DR; and Myeloid + Granulocytes using CD14, CD16, HLA-DR, CD11c, CD11b, CD38, CD24, and CD45RA.

Meta-clusters were manually annotated based on median marker-expression profiles and lineage consistency, yielding 27 final cell subtypes: 10 T-cell subtypes, 4 B-cell subtypes, 2 NK-cell subtypes, and 11 myeloid/granulocyte subtypes. Subtype names were fixed before downstream comparative analyses and used consistently throughout the study.

### Dimensionality reduction and compositional visualization

For sample-level visualization of immune composition, each sample was represented by a 27-dimensional vector corresponding to subtype proportions. Principal component analysis (PCA) was then performed on the resulting 45 x 27 sample-by-subtype frequency matrix to visualize global compositional relationships among samples.

For cell-level visualization, t-distributed stochastic neighbor embedding (tSNE) was computed using openTSNE in Python. Antibody-marker intensities, excluding DNA, cisplatin, bead/technical channels, and CD45, were arcsinh-transformed and reduced to 10 principal components. The global embedding was fit on a balanced subset of up to 5,000 cells per sample (perplexity = 30) and then applied to 9,107,515 CD45+ cells retained for downstream analysis. Lineage-specific embeddings were refit independently within each lineage using the same perplexity on lineage-specific subsets (up to 120,000 cells; NK cells, 106,718 cells) before transforming the retained cells from that lineage.

### Bulk monocyte HLA-DR intensity

To derive a simplified protein-level measure of monocyte HLA-DR expression independent of subtype annotation, we calculated bulk monocyte HLA-DR median fluorescence intensity (MFI) for each sample. This metric was defined as the median arcsinh-transformed HLA-DR signal among events assigned to the Myeloid + Granulocytes lineage with CD14 >0.5, thereby summarizing aggregate HLA-DR abundance across the monocyte-enriched compartment.

### Developmental immune-state modeling

To quantify disease-associated deviation from postnatal immune development, we constructed a reference immune-state axis using only the Birth and postnatal Control samples. For each sample, subtype proportions were standardized and represented in the 27-subtype compositional space. The developmental reference axis was then defined as the vector connecting the Birth centroid and the postnatal Control centroid, thereby capturing the dominant direction of maturational immune remodeling within the non-NEC reference set. Projection of each sample onto this axis yielded a continuous state-position score, with lower values corresponding to a more birth-like, myeloid-skewed configuration and higher values corresponding to a more postnatal control-like, lymphocyte-enriched configuration.

To account for postnatal developmental variation, state position was regressed against DOL using Birth and postnatal Control samples as the developmental reference. For each sample, the fitted value from this reference model represented the DOL-expected state position, and state displacement was calculated as the observed state position minus the fitted value.

### Immune network analysis

To evaluate whether NEC was associated not only with shifts in cell abundance but also with altered coordination among immune populations, we performed sample-level correlation-network analysis using the 27 annotated cell subtypes. For each study group, pairwise Spearman correlation coefficients were calculated across subtype proportions, generating a group-specific correlation matrix. Disease-associated changes in pairwise relationships were quantified as the difference in correlation coefficients between groups (for example, Delta rho NEC-Control = rhoNEC - rhoControl), and development-associated changes were analogously defined within the Birth-to-Control reference framework. Statistical significance of differences in correlation strength between groups was assessed using Fisher’s z-transformation, followed by Benjamini-Hochberg false discovery rate correction. To summarize the topological importance of each subtype within a given network, hub centrality was defined as the mean absolute correlation of that subtype with all other subtypes. Correlation sign reversals, defined as transitions from positive to negative correlations or vice versa between conditions, were extracted and used to identify the cell populations most centrally involved in network rewiring. Robustness of the network results was assessed by resampling infants with replacement within each group (2000 bootstrap replicates; percentile 95% confidence intervals and sign-consistency for the headline development-versus-disease coupling and for the highlighted edges) and by label permutation (2000 permutations; shuffling the three-way group labels to build a null distribution for the headline coupling, and shuffling Control/NEC labels to test whether the number of significantly rewired edges exceeded chance) (**Supplementary Table 17**).

### Analysis of publicly available intestinal transcriptomic datasets

To examine whether the dominant peripheral CyTOF signal mapped onto a related myeloid program in the diseased intestine, we analyzed two publicly available intestinal transcriptomic datasets: one neonatal NEC single-cell RNA-seq dataset (6) (https://doi.org/10.5281/zenodo.5813397) and one independent intestinal bulk RNA-seq dataset, GSE297483, from Lee et al. (12).

For the intestinal scRNA-seq analysis, monocyte/macrophage subsets were reanalyzed from the source dataset using the published processing framework of the original study (6). CyTOF-defined myeloid subtypes were then compared with scRNA-seq-derived intestinal monocyte states using a curated marker-to-gene mapping scheme: CD14 to CD14, CD16 to FCGR3A, HLA-DR to HLA-DRA, CD11c to ITGAX, CD11b to ITGAM, CD38 to CD38, and CD24 to CD24. For each CyTOF myeloid subtype, median arcsinh-transformed protein expression was compared with mean log-normalized gene expression in the corresponding scRNA-seq subclusters after feature-wise rescaling. Cross-platform concordance was quantified using Spearman correlation across shared protein-gene features. Differential abundance of intestinal monocyte subclusters between NEC and neonatal control samples was evaluated using the Mann-Whitney U test. To characterize the transcriptional program corresponding to the inflammatory intestinal monocyte state, gene set enrichment analysis was performed for the C2 subcluster versus the remaining myeloid compartment using fgsea with Gene Ontology Biological Process and KEGG gene sets obtained from MSigDB.

For the intestinal bulk RNA-seq analysis, the GSE297483 dataset described by Lee et al. (12) was used as an orthogonal tissue-level validation dataset and included control (n = 3), margin (n = 10), and lesion (n = 10) samples. To summarize MHC-II-associated antigen-presentation capacity, an HLA-DR/MHC-II composite score was calculated as the mean z-scored expression of HLA-DRA, HLA-DRB1, HLA-DPA1, CD74, CIITA, HLA-DPB1, and HLA-DQA1. Paired lesion-minus-margin differences for individual genes were assessed using the Wilcoxon signed-rank test. Pathway-level scores were then computed for curated inflammatory and immune-regulatory gene sets, including TNFa/NF-kB signaling, inflammatory response, IL-6/JAK-STAT3 signaling, IFN-gamma response, complement, hypoxia, reactive oxygen species, and MHC-II/antigen-presentation pathways, and compared between lesion and margin using paired Wilcoxon signed-rank testing.

### Software and statistical analysis

Primary analyses were performed in Python (version 3.10) and R (version 4.0). Continuous two-group comparisons were assessed using two-sided Mann-Whitney U or Wilcoxon rank-sum tests as appropriate, and comparisons involving more than two groups were assessed using Kruskal-Wallis tests followed by Dunn’s or pairwise Wilcoxon *post hoc* comparisons where appropriate. Associations between immune features and continuous clinical variables were evaluated using Spearman rank correlation; partial Spearman correlation adjusting for PMA was used for analyses designed to reduce confounding by developmental timing.

Differential abundance of immune cell subtypes was modeled separately for each of the 27 final subtypes using a negative-binomial generalized linear model on integer subtype event counts, with the log of total annotated CD45+ events per sample included as an offset. Within-infant dependence from serial sampling was handled using patient-clustered robust standard errors. Effect sizes are reported as log2 fold-changes with Wald 95% confidence intervals. Within each contrast, P values were adjusted across the 27 subtypes using the Benjamini-Hochberg false-discovery rate.

Prespecified sensitivity and robustness analyses included one-sample-per-infant analysis, adjustment for gestational age, PMA, and DOL, restriction to infants with gestational age <32 weeks, within-group outlier exclusion, leave-one-out analysis across NEC-at-onset samples, exclusion of very-low-count subtypes from the FDR family, equal-depth rarefaction to 44,578 CD45+ events per sample over 300 iterations, and a final clinical-core adjustment model incorporating perinatal, developmental, pre-sampling treatment, and infection covariates. Full model outputs are provided in **Supplementary Tables 10**-**14**.

ROC analysis of bulk monocyte HLA-DR MFI was treated as an exploratory within-cohort analysis rather than a clinical validation analysis; AUC values were estimated with nonparametric bootstrap 95% confidence intervals (5,000 resamples). Multiple-testing correction was applied according to analysis type: Bonferroni correction for subtype-level developmental correlation screening, Benjamini-Hochberg correction for subtype and network analyses, and Holm-Bonferroni correction for the remaining families of inferential tests unless otherwise stated. Two-sided adjusted P values <0.05 were considered statistically significant.

## Results

### Cohort characteristics and study design

**Figure 1:**
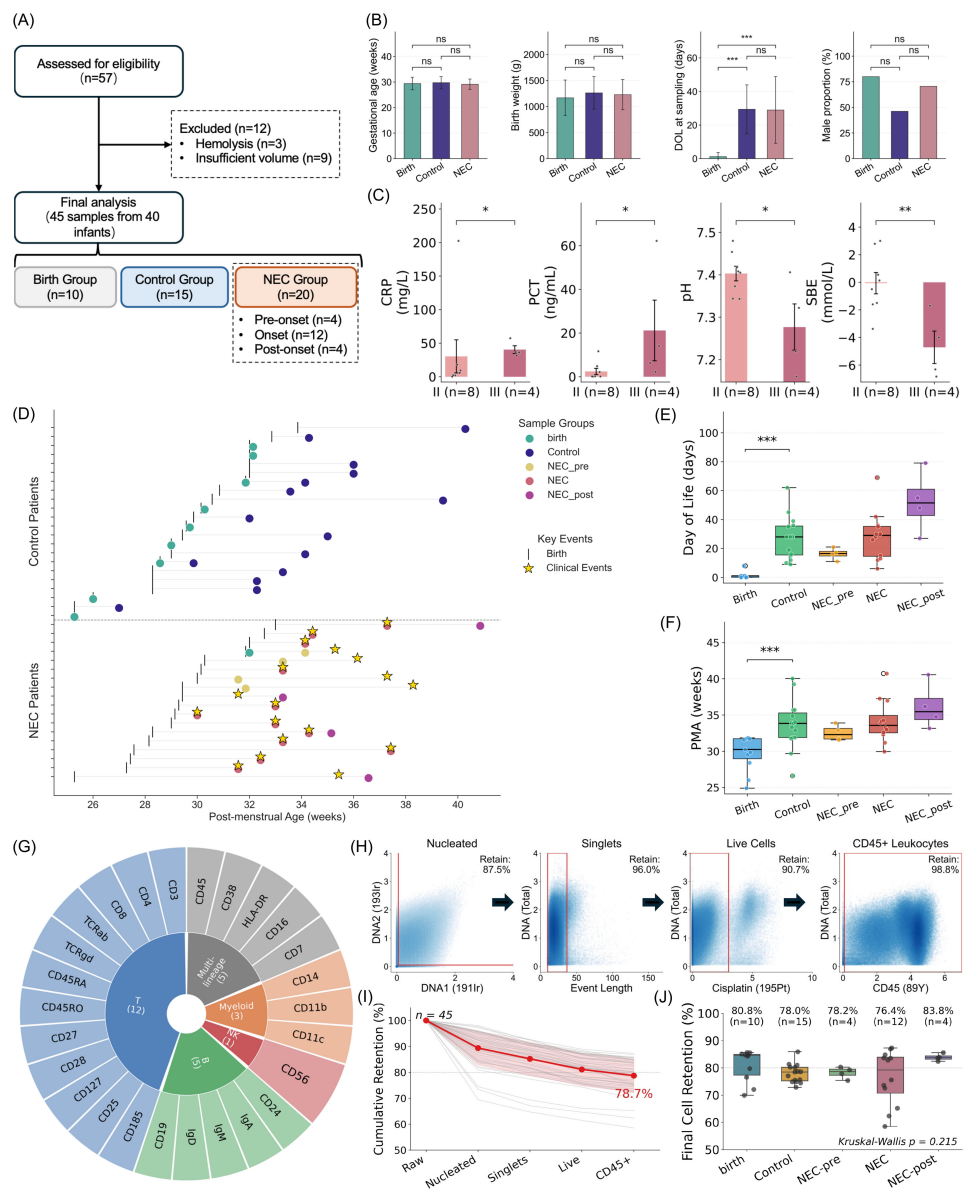
A quality-controlled neonatal CyTOF cohort enables immune profiling across NEC progression. **(A)** Flow diagram of cohort inclusion and exclusion. Fifty-seven samples were assessed for eligibility; 12 were excluded because of hemolysis or insufficient blood volume, leaving 45 samples from 40 infants for final analysis. The final cohort comprised Birth, Control, NEC-pre, NEC-at-onset, and NEC-post samples. **(B)** Baseline and sampling characteristics across Birth, Control, and NEC groups, including gestational age, birth weight, day of life at sampling, and male proportion. Bars show group summaries with pairwise statistical annotations. **(C)** Representative onset clinical variables comparing Bell stage II and stage III NEC, including C-reactive protein, procalcitonin, pH, and standard base excess. **(D)** Per-patient sampling timeline plotted by postmenstrual age, with sample groups and key clinical events indicated. **(E, F)** Day of life and postmenstrual age distributions across the five sample groups. **(G)** 26-marker antibody panel design summarized by lineage and functional marker category. **(H)** Representative gating workflow for the OPTIMAL preprocessing pipeline, including nucleated cells, singlets, live cells, and CD45+ leukocytes. **(I)** Cumulative retention across preprocessing steps for all valid samples. **(J)** Final CD45+ cell retention by group. Unless otherwise indicated, continuous variables were compared using nonparametric tests and multiple-group comparisons were adjusted as appropriate; *, P < 0.05; **, P < 0.01; ***, P < 0.001; ns, not significant. CD, cluster of differentiation; CRP, C-reactive protein; DOL, day of life; NEC, necrotizing enterocolitis; PCT, procalcitonin; PMA, postmenstrual age; SBE, standard base excess.

We profiled peripheral blood immunity in preterm neonates with and without NEC using a 26-marker CyTOF panel spanning the major lymphoid and myeloid compartments. Forty-five samples from 40 unique infants passed quality control and were included in the final analysis (**Figure 1A**; **Supplementary Table 1**). These samples represented five predefined clinical groups: Birth (n = 10), Control (n = 15), NEC-pre (n = 4), NEC at onset (n = 12), and NEC-post (n = 4). At the patient level, gestational age, birth weight, sex, and key pre-sampling clinical and treatment factors were broadly comparable across groups (**Figures 1B, D, E**; **Supplementary Tables 2**, **3**). Among NEC cases sampled at diagnosis, Stage III infants showed a more severe inflammatory-metabolic profile than Stage II, with higher CRP and PCT and worse acid-base status (lower pH and SBE) (**Figure 1C**; **Supplementary Figure 1A**). Although DOL and postmenstrual age (PMA) differed across the five sampling groups overall (**Figure 1D**), DOL and PMA were comparable between postnatal Control and NEC-at-onset samples (**Figures 1E, F**). Compared with Control, NEC-at-onset samples did not differ significantly in gestational age, birth weight, sex, major perinatal variables, or most pre-sampling treatment and microbiology indicators after Holm correction; pre-sampling antibiotic duration and nil-per-os duration were accounted for in sensitivity and clinical-core analyses (**Supplementary Figure 1C**; **Supplementary Table 3**). Following the four-step preprocessing workflow, the mean CD45+ acquisition yield was 78.7% (median, 79.7%), yielding 12.66 million CD45+ leukocytes for downstream analysis, with a median of 310,740 cells per sample (**Figures 1G–J**; **Supplementary Table 1**).

### Peripheral immune profiles of preterm neonates follow a postnatal maturational trajectory

**Figure 2:**
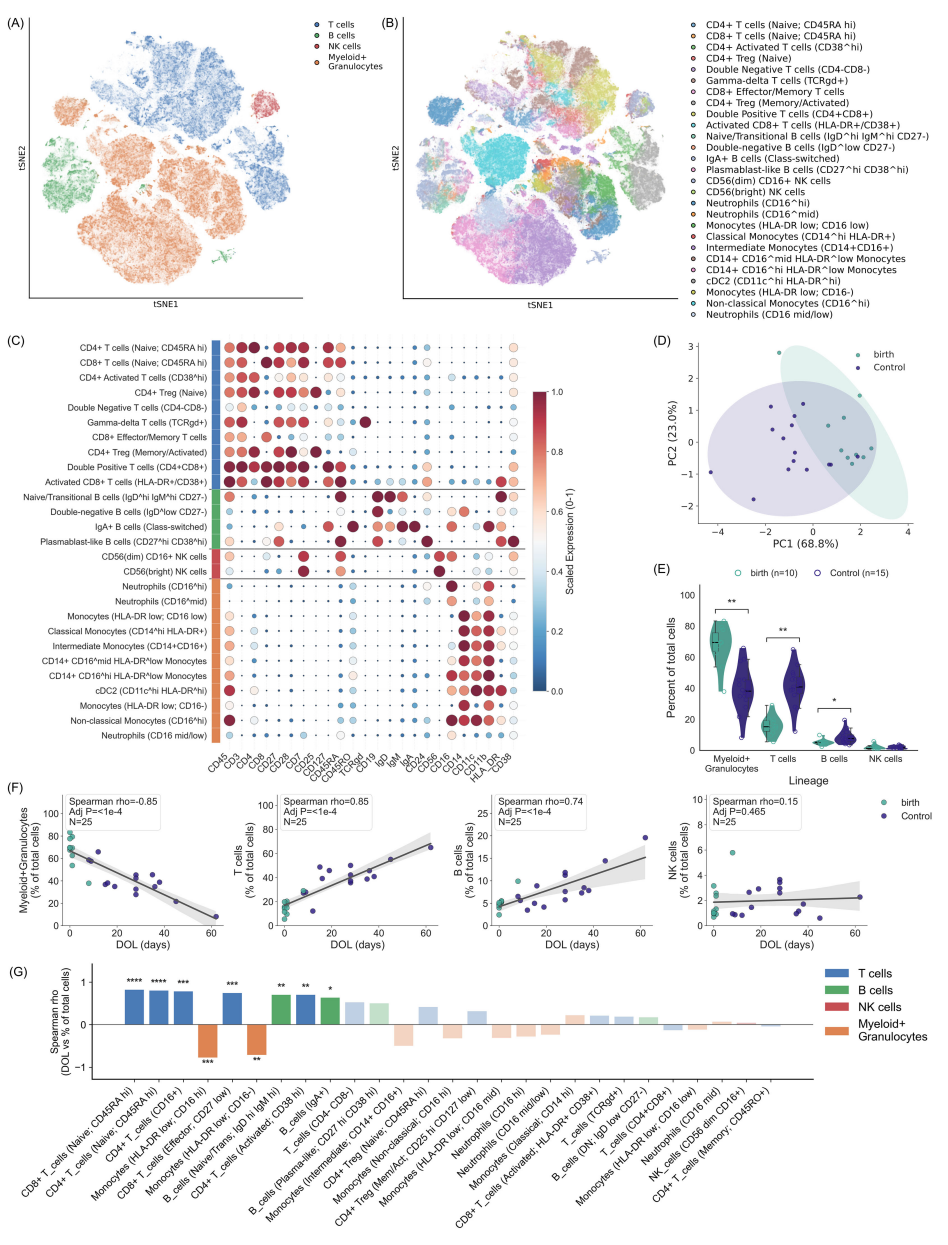
A high-dimensional CyTOF atlas resolves neonatal immune lineages and postnatal maturation. **(A)** tSNE embedding of all OPTIMAL-preprocessed CD45+ cells colored by four major lineages. Myeloid and granulocyte populations are shown together as Myeloid+Granulocytes. **(B)** tSNE embedding colored by the final annotated immune cell subtypes. **(C)** Marker-expression dot plot for the final annotated subtypes. Dot color and size represent scaled marker expression. **(D)** Principal-component analysis of major-lineage proportions in Birth and Control samples, showing 95% confidence ellipses. **(E)** Major-lineage proportions in Birth versus Control samples. **(F)** Associations between day of life and major-lineage proportions in Birth and Control samples. Lines indicate fitted trends with 95% confidence bands; Spearman correlations and adjusted P values are shown. **(G)** Cell-subtype associations with day of life, summarized by Spearman rho and colored by lineage. Asterisks denote statistical significance after multiple-testing correction. CD, cluster of differentiation; CyTOF, cytometry by time-of-flight; DOL, day of life; NK, natural killer; PCA, principal-component analysis; tSNE, t-distributed stochastic neighbor embedding.

Global tSNE resolved neonatal peripheral blood into four major immune compartments — T cells, B cells, NK cells, and Myeloid + Granulocytes — within which 27 cell subtypes could be robustly defined, each supported by a distinct marker-expression profile (**Figures 2A–C**; **Supplementary Table 4**; **Supplementary Figure 2**).

The overall immune profiles showed significant separation between birth and postnatal samples of non-NEC preterm neonates (PERMANOVA: R² = 49.6%, P = 0.0001; **Figure 2D**). This was marked by a significant difference in lineage composition: Birth samples were enriched for myeloid cells, whereas postnatal samples showed higher T-cell and B-cell proportions (**Figure 2E**; **Supplementary Table 5**). Similarly, the proportion of myeloid cells declined with increasing DOL, whereas T-cell and B-cell proportions rose (**Figure 2F**). At the subtype level, the proportions of activated T-cell subsets (particularly activated CD4+ and CD8+ T cells) showed strong positive associations with DOL, whereas the proportions of HLA-DR-low monocytes showed strong negative associations with DOL (**Figure 2G**; **Supplementary Figure 3**; **Supplementary Table 6**). Overall, peripheral immune profiles of preterm neonates followed a postnatal transition from a myeloid-enriched profile toward a lymphocyte-enriched profile.

### NEC onset is associated with HLA-DR-low monocyte-centered displacement from the developmental immune trajectory

**Figure 3:**
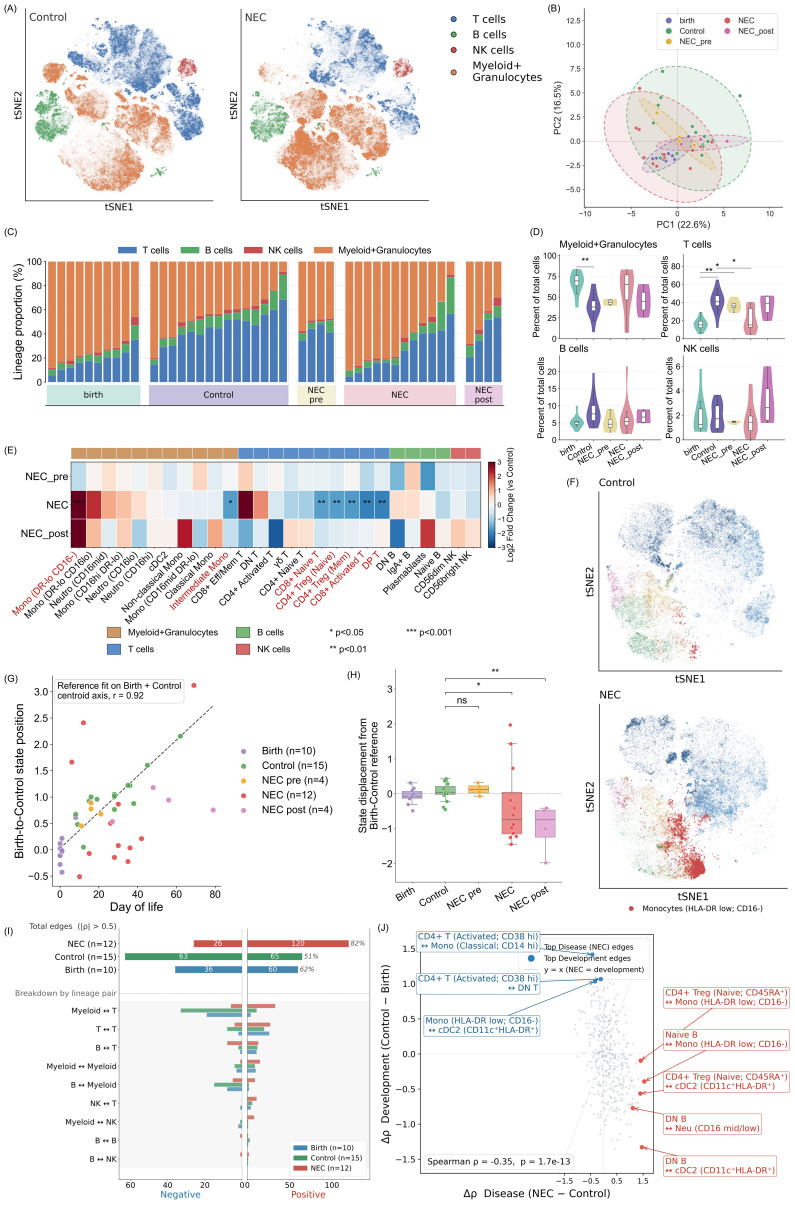
NEC onset displaces the neonatal immune landscape toward an HLA-DR-low monocyte state. **(A)** Global tSNE projections comparing Control and NEC samples at the lineage level. **(B)** Principal-component analysis of immune-cell composition across Birth, Control, NEC-pre, NEC, and NEC-post groups, with 95% confidence ellipses. **(C)** Major-lineage proportions across individual samples ordered by group. **(D)** Group distributions of major-lineage proportions. **(E)** Subtype-level log2 fold changes versus Control across NEC-pre, NEC, and NEC-post samples, displayed as a heatmap with lineage-colored column annotations; asterisks indicate statistically significant differences after multiple-testing correction. **(F)** tSNE projections highlighting HLA-DR-low CD16-negative monocytes in Control and NEC samples. **(G)** Birth-to-Control immune-state position plotted against day of life; the dashed line indicates the reference fit derived from Birth and Control samples. **(H)** State displacement, defined as observed minus DOL-expected state position, across the five groups. **(I)** Directional breakdown of network-edge signs across Birth, Control, and NEC samples. **(J)** Edge-level comparison of development-associated changes and NEC-associated changes in pairwise immune-cell correlations; highlighted edges indicate representative development-associated and NEC-associated rewiring events. The global edge-level association is shown descriptively, while specific rewired edges were evaluated using Fisher r-to-z testing with false-discovery-rate correction. CD, cluster of differentiation; DOL, day of life; HLA-DR, human leukocyte antigen-DR; NEC, necrotizing enterocolitis; PCA, principal-component analysis; tSNE, t-distributed stochastic neighbor embedding.

We next evaluated NEC samples against the developmental reference framework defined by the Birth and Control groups. At the global level, tSNE showed clear redistribution of the peripheral immune landscape in NEC relative to Control, characterized by expansion of the Myeloid and Granulocytes compartment together with contraction of the T-cell compartment (**Figure 3A**; **Supplementary Figure 4A**). PCA also placed NEC samples apart from the Birth-to-Control reference space, supporting a broad compositional shift upon NEC onset (**Figure 3B**). Consistent with these ordination patterns, lineage-level quantification identified myeloid expansion and T-cell contraction as the dominant axis of NEC-associated deviation, whereas changes in B-cell and NK-cell abundance were comparatively modest (**Figures 3C, D**; **Supplementary Table 5**).

At subtype resolution, this shift was concentrated in a limited set of populations rather than distributed broadly across the immune compartment. The full distribution of all 27 subtype frequencies across sample groups is shown in **Supplementary Figure 6**. The most prominent NEC-associated change was a marked expansion of HLA-DR-low CD16-negative monocytes, which increased from 0.005% in Controls to 1.27% in NEC-at-onset samples (negative-binomial GLM with patient-clustered robust inference: log2FC = 7.88, FDR = 2.1 x 10^-8), the largest effect across all 27 subtypes. This expansion remained positive and statistically significant across prespecified sensitivity and robustness analyses, including one-sample-per-infant analysis, developmental covariate adjustment, restriction to very-preterm infants, within-group outlier exclusion, leave-one-out analysis, low-count-subtype exclusion, equal-depth rarefaction, and the final clinical-core adjustment (all FDR < 0.05 where applicable; rarefaction log2FC = 7.92, 95% interval 7.54-8.36) (**Figures 3E, F**; **Supplementary Figure 5**; **Supplementary Tables 10**-**12**, **14**). This population showed little change in NEC-pre, peaked at clinical onset, and remained only partially reduced in NEC-post samples (**Figure 3E**; **Supplementary Tables 7**, **9**). In parallel, several T-cell subsets contracted in NEC, including CD8+ Naive T cells, CD4+ Treg Naive cells, CD4+ Treg Memory/Activated cells, Double Positive T cells, and Activated CD8+ T cells (**Figure 3E**; **Supplementary Table 8**). Together, these data identify a coupled axis of HLA-DR-low monocyte expansion and naive/regulatory T-cell depletion as the principal cellular feature associated with NEC onset.

Because HLA-DR-low CD16-negative monocytes were rare in postnatal Controls, we tested whether the effect depended on low event counts or acquisition depth. In NEC-at-onset samples this subtype was consistently measurable (median 760 events per sample; 12/12 samples >=20 events), whereas its near-absence in Controls (median 5 events) was more consistent with biological scarcity than acquisition failure. The NEC enrichment remained evident on a frequency basis, was not explained by total CD45+ acquisition depth, and persisted under equal-depth rarefaction to 44,578 CD45+ events per sample over 300 iterations (**Supplementary Figures 5B, C**; **Supplementary Tables 13**, **14**).

To determine whether the NEC-associated shift could be explained by postnatal timing alone, we projected all samples onto the Birth-to-Control reference axis and modeled state position as a function of DOL within the Birth plus Control reference set. State position increased strongly with DOL (r = 0.92, P = 6.4 x 10^-11), consistent with progression along the postnatal developmental reference (**Figure 3G**). After subtracting the DOL-expected state position, NEC-pre samples remained close to postnatal Controls (median, +0.12; P = 0.665), whereas NEC-at-onset samples showed significant negative state displacement (median, -0.74 versus +0.03 in Controls; P = 0.048). NEC-post samples also remained negatively displaced (median, -0.75; P = 0.001) (**Figure 3H**; **Supplementary Tables 15**, **16**). Together with the lineage- and subtype-level results, these findings indicate that NEC at onset was associated with displacement below the DOL-expected developmental immune state and with a shift toward the myeloid/HLA-DR-low pole of the reference trajectory.

Network analysis showed that this deviation involved not only altered cellular composition but also reorganization of inter-cellular coordination (**Figure 3I**). Using a |rho| >0.5 visualization threshold, positive and negative correlations among immune cell subsets were relatively balanced in Controls (50.4% and 49.6%, respectively), whereas positive edges predominated in NEC (82.2%) (**Figure 3I**), consistent with loss of the counterbalancing relationships that normally structure the developing peripheral immune system. Development-associated and disease-associated changes in pairwise correlations were inversely related across edges (Spearman rho = -0.35, bootstrap 95% CI [-0.59, -0.15], sign-consistent in 100% of resamples; **Figure 3J**; **Supplementary Table 17**), but this global anti-correlation was not beyond the label-permutation null (permutation P = 0.58) and is therefore interpreted as descriptive rather than as an independent significance test. The disease-associated rewiring itself was unlikely under Control/NEC label permutation: 8 edges differed significantly between NEC and Control (Fisher r-to-z, Benjamini-Hochberg FDR <0.05) versus approximately 0.8 expected by chance (permutation P = 0.019; **Supplementary Table 17**). The strongest sign reversals were centered on HLA-DR-low monocytes and were stable under within-group bootstrap resampling, including Naive/Transitional B cells versus HLA-DR-low monocytes (rho from -0.70 to +0.85; Delta rho = 1.55, bootstrap 95% CI [0.94, 1.80]; adjusted P = 7.0 x 10^-4) and Naive Treg cells versus HLA-DR-low monocytes (rho from -0.68 to +0.73; Delta rho = 1.41, bootstrap 95% CI [0.74, 1.79]; adjusted P = 8.5 x 10^-3) (**Figure 3J**; **Supplementary Figure 7**; **Supplementary Table 17**). Taken together, these analyses show that NEC onset is associated with coordinated displacement away from the developmental immune trajectory, centered on expansion of HLA-DR-low monocytes and accompanied by contraction of regulatory/naive T-cell compartments and disease-associated rewiring of specific immune-cell correlations.

### Independent intestinal transcriptomic datasets link peripheral HLA-DR-low monocytes to an inflammatory, MHC-II-suppressed myeloid state in NEC

**Figure 4:**
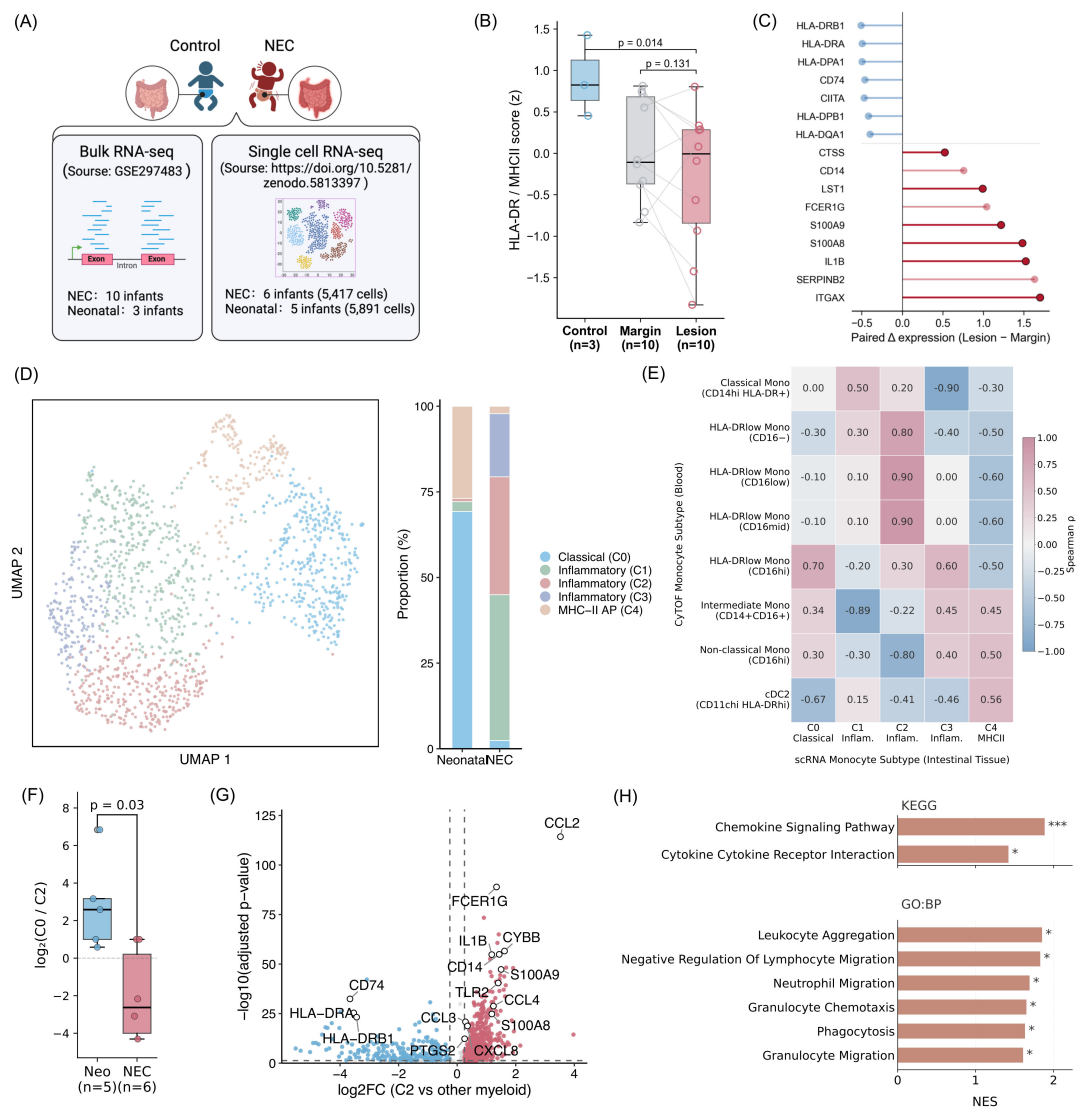
Peripheral HLA-DR-low monocytes align with inflammatory MHC-II-suppressed intestinal myeloid states. **(A)** Overview of external intestinal transcriptomic validation datasets used for cross-tissue comparison, including bulk RNA-seq and single-cell RNA-seq cohorts. **(B)** Bulk RNA-seq comparison of HLA-DR/MHC-II module activity across Control, margin, and lesion intestinal tissue. Paired lines indicate matched samples when available. **(C)** Paired lesion-minus-margin expression changes for representative antigen-presentation, inflammatory, and myeloid-associated genes. **(D)** Single-cell RNA-seq UMAP of intestinal monocyte/macrophage populations with subtype proportions in neonatal and NEC samples. **(E)** Correlation heatmap comparing CyTOF monocyte subtypes with intestinal single-cell monocyte/macrophage subtypes. **(F)** Log2(C0/C2) ratio comparing classical and inflammatory intestinal monocyte states in neonatal and NEC samples. **(G)** Differential-expression volcano plot comparing the NEC-associated intestinal myeloid subtype with other myeloid cells, highlighting inflammatory and antigen-presentation-related genes. **(H)** Gene-set enrichment results for the NEC-associated intestinal myeloid subtype, including KEGG and Gene Ontology biological-process terms. Statistical tests are indicated in each panel; asterisks denote nominal or adjusted significance as displayed. BP, biological process; CD, cluster of differentiation; CyTOF, cytometry by time-of-flight; HLA-DR, human leukocyte antigen-DR; KEGG, Kyoto Encyclopedia of Genes and Genomes; MHC-II, major histocompatibility complex class II; NEC, necrotizing enterocolitis; RNA-seq, RNA sequencing; scRNA-seq, single-cell RNA sequencing; UMAP, uniform manifold approximation and projection.

Having identified expansion of HLA-DR-low monocytes as the dominant peripheral immune alteration associated with NEC, we next asked whether this blood-derived signal corresponded to a related myeloid state in the diseased intestine. To address this, we examined two publicly available intestinal transcriptomic datasets; sample sizes, platforms, and data sources are summarized in **Figure 4A**.

We first assessed tissue-level changes using an independent intestinal bulk RNA-seq cohort. Compared with control tissue, NEC lesion samples exhibited significantly lower HLA-DR/MHC-II composite scores, with margin samples occupying an intermediate position (P = 0.014 for control versus lesion) (**Figure 4B**). Paired margin-to-lesion comparisons within the same patients showed coordinated downregulation of MHC-II-related genes, including HLA-DRA, HLA-DRB1, CD74, and CIITA, together with upregulation of inflammatory genes such as S100A8/A9, IL1B, and CD14 (**Figure 4C**).

We next used neonatal NEC intestinal scRNA-seq to resolve monocyte states at single-cell resolution. Monocytes segregated into five subclusters (C0-C4) spanning classical (C0), inflammatory (C1-C3), and MHC-II/antigen-presentation (C4) states (**Figure 4D**). Cross-platform mapping showed that CyTOF-defined HLA-DR-low monocytes aligned most strongly with the inflammatory intestinal subclusters, with the highest correspondence observed for C2, an inflammatory/MDSC-like population, whereas classical monocytes showed an opposing relationship with this state (**Figure 4E**; **Supplementary Tables 19**, **20**). This mapping was accompanied by a compositional shift within the intestinal dataset itself: C2 was relatively enriched in NEC tissue, resulting in a significantly reduced log2(C0/C2) ratio compared with neonatal controls (P = 0.03) (**Figure 4F**). At the transcriptional level, C2 was characterized by increased expression of inflammatory genes, including CCL2, S100A8/A9, IL1B, and CD14, together with reduced expression of antigen-presentation genes such as HLA-DRA, CD74, and CIITA (**Figure 4G**). Gene set enrichment analysis further showed enrichment of chemokine signaling, cytokine-cytokine receptor interaction, neutrophil/granulocyte migration, and phagocytosis pathways in this cluster (**Figure 4H**).

Together, these bulk and single-cell intestinal datasets showed a concordant pattern to the CyTOF signal in blood: inflammatory myeloid activation occurring together with reduced HLA-DR/MHC-II-associated antigen-presentation capacity. This supports the interpretation that the peripheral HLA-DR-low monocyte signal reflects a broader NEC-associated myeloid program marked by concurrent inflammatory activation and attenuation of MHC-II-related antigen-presentation machinery.

### Bulk monocyte HLA-DR expression reflects the severity-linked NEC monocyte state and provides an exploratory within-cohort readout

**Figure 5:**
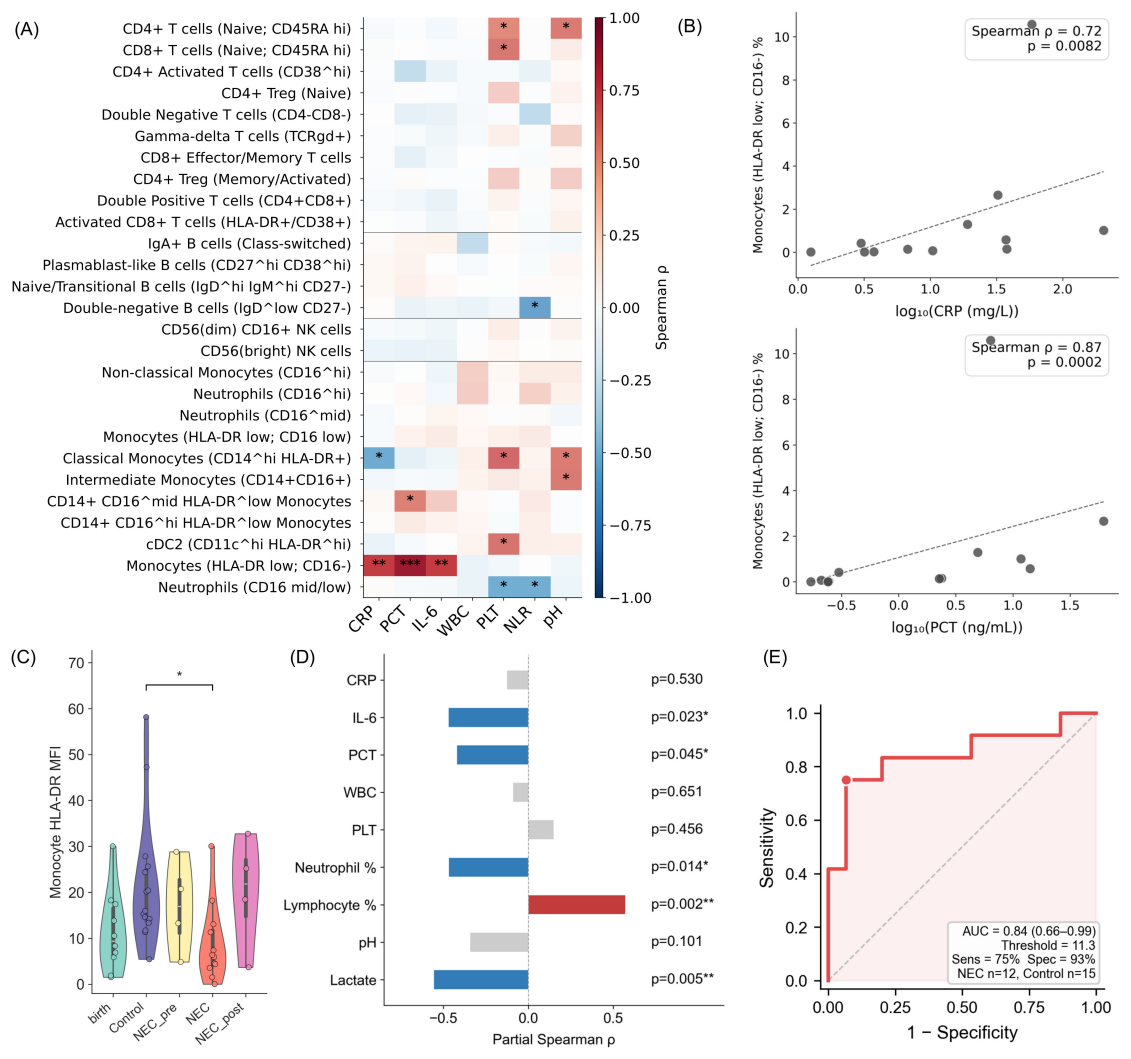
Monocyte HLA-DR suppression tracks NEC inflammatory severity and provides an exploratory within-cohort readout. **(A)** Heatmap of Spearman correlations between immune cell subtypes and clinical laboratory variables at NEC onset, including inflammatory, hematologic, acid-base, and metabolic indicators. Cell colors represent Spearman rho, and asterisks indicate statistically significant associations after multiple-testing correction. **(B)** Scatter plots showing associations between HLA-DR-low CD16-negative monocyte frequency and C-reactive protein or procalcitonin. Dashed lines indicate fitted trends; Spearman correlations and P values are shown. **(C)** Monocyte HLA-DR median fluorescence intensity across sample groups. **(D)** Partial Spearman correlations between monocyte HLA-DR expression and clinical variables after adjustment for relevant covariates, with P values shown at right. **(E)** Exploratory receiver-operating-characteristic analysis of monocyte HLA-DR expression comparing NEC-at-onset and postnatal Control samples. The plot reports AUC = 0.84 with bootstrap 95% confidence interval 0.65-0.99. Asterisks denote statistical significance as displayed. AUC, area under the receiver-operating-characteristic curve; CD, cluster of differentiation; CRP, C-reactive protein; HLA-DR, human leukocyte antigen-DR; IL, interleukin; MFI, median fluorescence intensity; NEC, necrotizing enterocolitis; NLR, neutrophil-to-lymphocyte ratio; PCT, procalcitonin; PLT, platelet count; ROC, receiver-operating characteristic; WBC, white blood cell count.

We next asked whether the HLA-DR-low monocyte axis identified above was linked to contemporaneous clinical severity within the NEC cohort. Correlation analysis using same-day laboratory measurements showed that HLA-DR-low CD16-negative monocytes were the cell subtype most consistently aligned with inflammatory burden. Their abundance correlated positively with CRP (rho = 0.72, P = 0.008) and PCT (rho = 0.87, P = 0.0002), and also showed positive associations with IL-6, WBC, and neutrophil-to-lymphocyte ratio (NLR), while correlating inversely with pH (**Figures 5A, B**; scatter plots shown on log10 scale). By contrast, Classical Monocytes (CD14hi HLA-DR+) showed the opposite pattern, including a negative correlation with CRP, indicating reciprocal behavior of the HLA-DR-high and HLA-DR-low monocyte compartments (**Figure 5A**; **Supplementary Table 18**). This reciprocal monocyte axis was also evident across NEC severity strata, with the higher-severity subgroup showing higher HLA-DR-low monocyte proportions and lower Classical Monocyte proportions than the lower-severity subgroup (**Supplementary Figure 4C**). Together, these findings place HLA-DR-low monocyte expansion within the clinically more inflamed end of the NEC immune spectrum.

Because the proportion of HLA-DR-low monocytes repeatedly emerged as the dominant disease-linked signal, we next asked whether a simplified protein-level metric could summarize the same signal. We therefore quantified bulk monocyte HLA-DR median fluorescence intensity (MFI) across samples. Monocyte HLA-DR MFI was markedly reduced in NEC-at-onset samples relative to postnatal Controls, with suppression already evident in NEC-pre samples and persisting into NEC-post samples (**Figure 5C**). PMA-adjusted correlation analyses showed that lower monocyte HLA-DR MFI remained significantly associated with higher IL-6 (P = 0.023), higher PCT (P = 0.045), higher neutrophil proportion (P = 0.014), lower lymphocyte proportion (P = 0.002), and higher lactate (P = 0.005) (**Figure 5D**), indicating that reduced HLA-DR expression tracked inflammatory and metabolic severity independently of developmental timing. Thus, bulk monocyte HLA-DR MFI functioned as an aggregate protein-level readout of the NEC-associated HLA-DR-low monocyte-centered state.

Finally, we evaluated this simplified metric in an exploratory within-cohort ROC analysis comparing NEC-at-onset samples with postnatal Controls. Monocyte HLA-DR MFI yielded an AUC of 0.84 (bootstrap 95% CI 0.65-0.99; Mann-Whitney P = 0.0027; **Figure 5E**). These data indicate that monocyte HLA-DR expression reflects the dominant NEC-associated monocyte perturbation at the protein level and provides a compact readout associated with inflammatory severity in this dataset; they should not be interpreted as validation of a standalone clinical test.

## Discussion

By combining peripheral CyTOF and intestinal transcriptomic analyses, our findings suggest that NEC is associated with substantial alterations in both peripheral and intestinal immune profiles. In control preterm infants, peripheral immunity followed a coherent myeloid-to-lymphocyte maturational trajectory. NEC onset disrupted this trajectory and shifted the immune landscape into an HLA-DR-low, monocyte-dominant state accompanied by contraction of naive and regulatory T-cell populations and loss of the balanced intercellular architecture that normally organizes the developing immune system. Taken together, these findings identify HLA-DR-low monocyte-centered reprogramming as the dominant feature of NEC-associated systemic immune dysregulation in this cohort, which may serve as a mechanistically anchored candidate readout for immune monitoring in preterm infants with NEC.

Recent high-dimensional, single-cell, and targeted studies have begun to define key immune components of NEC, including systemic NK-cell activation, inflammatory intestinal macrophage states, altered regulatory T-cell biology, trafficking monocytes, and a broader contribution of myeloid dysregulation to disease pathogenesis (5–8, 13, 14). Our study extends these observations by placing them within a developmental reference framework. Anchored to a birth-to-postnatal remodeling trajectory (9), our data showed that NEC-associated immune remodeling was best captured not as diffuse immune activation, but as coordinated deviation toward an HLA-DR-low CD16-negative monocyte state, identifying this population as the principal peripheral axis of disease-associated displacement.

This interpretation connects NEC to a broader critical-care paradigm while preserving neonatal specificity. In adult sepsis, reduced monocyte HLA-DR is a well-established marker of immunoparalysis and adverse outcome (15, 16), and has been linked to dysfunctional monocyte reprogramming with attenuated antigen presentation and altered inflammatory responsiveness (16, 17). Importantly, monocyte HLA-DR has also functioned as a pharmacodynamically informative immune readout in GM-CSF-based immune restoration studies (18). In preterm infants, however, low monocyte HLA-DR should be interpreted more carefully in developmental context, because reduced expression is already a feature of baseline immune immaturity in very low birth weight infants (19) and has recently been identified as part of the sepsis immune signature in very preterm neonates (20). These findings suggest that NEC does not introduce an entirely novel immune state, but is associated with displacement toward an exaggerated, disease-associated state along a developmentally relevant myeloid axis.

The coordinated contraction of naive and regulatory T-cell compartments and the erosion of balanced cross-lineage correlations support structured immune remodeling rather than isolated abundance changes. The conceptual overlap with physiologic neonatal myeloid-derived suppressor cell-like programs is noteworthy (10), but the magnitude, timing, and network centrality of HLA-DR-low monocyte expansion in NEC argue against a purely homeostatic interpretation. One plausible framework is disease-triggered emergency myeloid skewing, analogous to the emergency granulopoiesis described in severe systemic inflammation and sepsis (21), although our data do not directly establish mechanism.

Importantly, the peripheral HLA-DR-low monocyte state also showed concordance with intestinal myeloid alterations, aligning with inflammatory intestinal myeloid states in scRNA-seq (6), while intestinal lesion transcriptomes showed concurrent inflammatory activation and selective suppression of MHC-II-related programs (12). This dual phenotype resembles myeloid states described in critical illness (17, 21) and is consistent with a shared blood-to-gut disease-associated myeloid program, while not proving a causal relationship between circulating and intestinal compartments. From a translational perspective, monocyte HLA-DR is of interest because it summarizes a systems-level phenotype as a measurable protein-level readout, rather than replacing high-dimensional profiling. In adult and pediatric critical care, monocyte HLA-DR has become a tractable indicator of immune competence and outcome risk (22–24), and in our cohort lower monocyte HLA-DR tracked inflammatory severity and showed exploratory within-cohort ROC performance. These observations support monocyte HLA-DR as a mechanistically grounded candidate immune-monitoring readout, rather than a clinically validated standalone test.

This study has several limitations. The cohort was single-center and modest in size, with small NEC-pre and NEC-post groups and partial repeated sampling, limiting patient-level temporal inference despite patient-clustered inference and one-sample-per-infant sensitivity analyses. NEC is clinically heterogeneous and multifactorial, and although we reviewed pre-sampling clinical exposures and performed developmental, treatment, restricted-cohort, and clinical-core adjustments, residual confounding cannot be fully excluded. Blood was obtained at routine clinical sampling times, and NEC-at-onset sampling coincided with standard-of-care evaluation at diagnosis rather than protocolized research-only time points. Exact per-sample blood volumes were not retained, although all valid samples were within the 300–500 uL range; sample-specific total nucleated-cell counts were not retained in the finalized analysis dataset, and exact per-sample processing times were not recorded beyond the protocolized 30 min to 1 h window, so residual processing-related variation cannot be fully excluded. CyTOF defined phenotype rather than function, so the biological role of HLA-DR-low monocytes cannot be established from these data alone. The HLA-DR-low CD16-negative monocyte population was rare in controls; rare-cell and rarefaction analyses support the robustness of the NEC enrichment, but external replication remains important. Finally, the ROC analysis of monocyte HLA-DR should be regarded as exploratory within-cohort evidence and requires independent validation before clinical use.

## Conclusion

NEC was associated with developmentally discordant systemic immune remodeling centered on HLA-DR-low CD16-negative monocytes. This monocyte-centered program was associated with peripheral immune displacement, disease-specific immune-correlation rewiring, and concordant inflammatory MHC-II-suppressed intestinal myeloid signatures. Monocyte HLA-DR therefore provides a biologically anchored candidate readout for immune monitoring, warranting external validation in larger preterm cohorts.

## Data Availability

The anonymized raw CyTOF FCS files and figure source data supporting this study are openly available in Zenodo at https://doi.org/10.5281/zenodo.21231338. The intestinal scRNA-seq dataset is available at https://doi.org/10.5281/zenodo.5813397. The intestinal bulk RNA-seq dataset is available from GEO under accession GSE297483.
