# Supplementary material for: Peripheral immune profiling identifies developmental immune displacement into an HLA-DR-low monocyte state in necrotizing enterocolitis
Source: Front Immunol. 2026 Jul 21;17:1868823. doi: 10.3389/fimmu.2026.1868823 (PMC13433625; doi:10.3389/fimmu.2026.1868823)
Supplement: Supplementary file 1 [file DataSheet1.pdf]

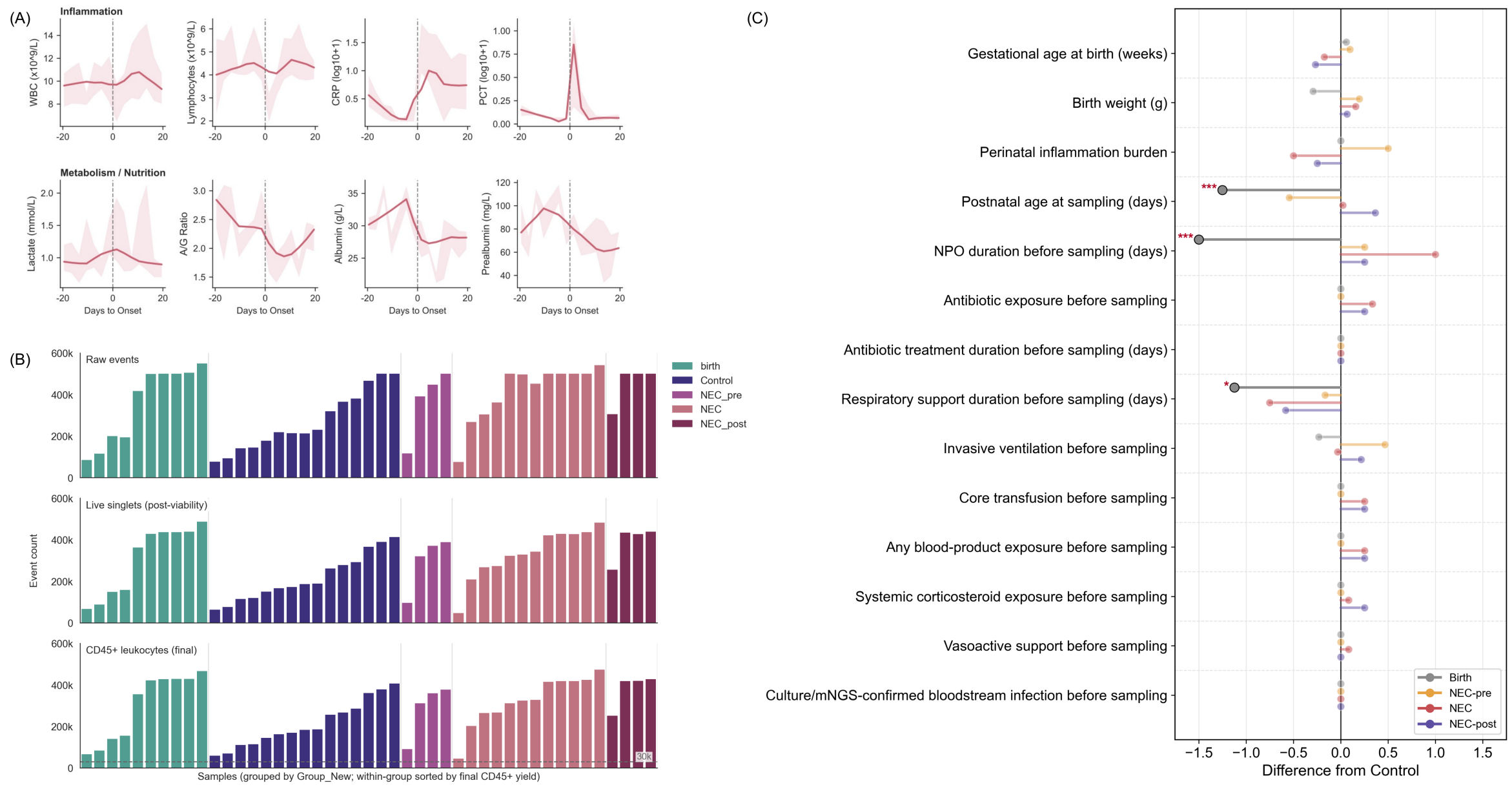

Figure S1. Sample-level quality control and key clinical-factor comparability.

(A) Temporal trajectories of representative clinical laboratory indices around NEC onset, including inflammatory and metabolic/nutritional variables. Lines indicate smoothed trends with shaded uncertainty bands; the vertical dashed line marks NEC onset. (B) Per-sample event yields across preprocessing workflow, including raw acquired events, live singlets after post-viability gating, and final CD45+ leukocyte events. Samples are grouped by clinical category and ordered within each group by final CD45+ yield. (C) Group differences in key pre-sampling clinical and treatment factors relative to Control.

Continuous variables are shown as median differences and binary variables as prevalence differences; treatment, microbiology, and exposure variables are restricted to the period before blood sampling. Pairwise comparisons versus Control were adjusted using Holm correction, with significance indicated directly on the plot.

Abbreviations: A/G, albumin-to-globulin ratio; ALB, albumin; CD, cluster of differentiation; CRP, C-reactive protein; mNGS, metagenomic next-generation sequencing; NEC, necrotizing enterocolitis; NLR, neutrophil-to-lymphocyte ratio; NPO, nil per os; PCT, procalcitonin; WBC, white blood cell count.

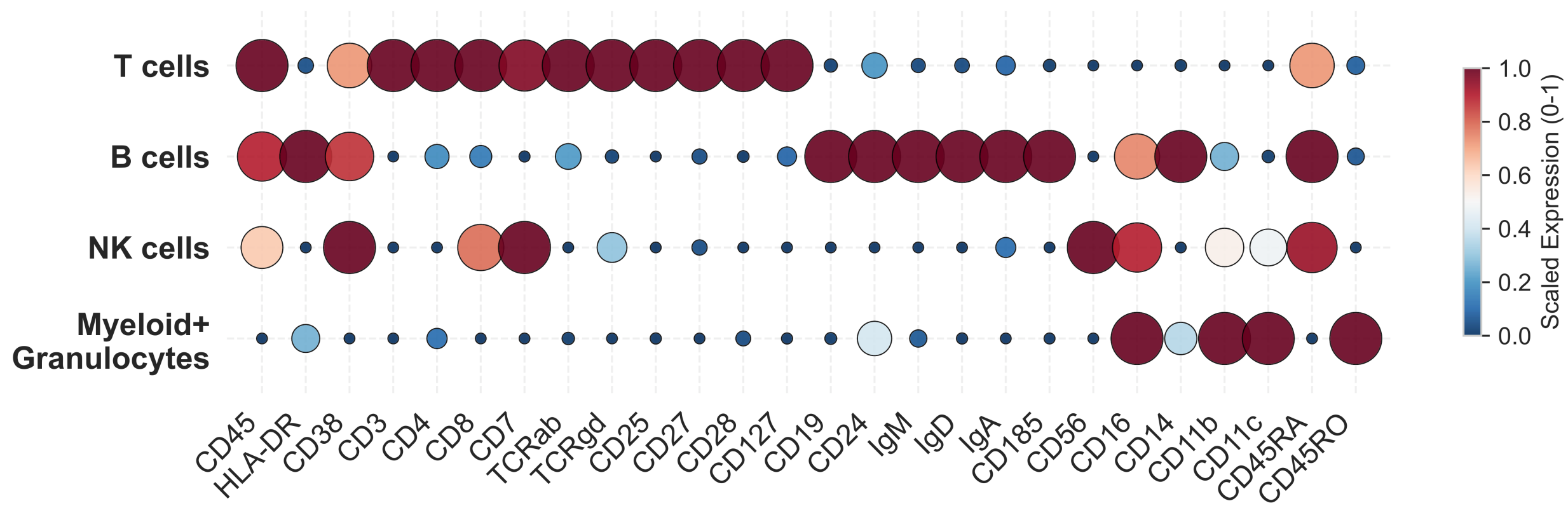

Figure S2. Marker-expression profile of the four major immune lineages.

Dot plot showing scaled median marker expression for the four major immune lineages: T cells, B cells, NK cells, and Myeloid+Granulocytes. Dot color and size both represent scaled expression from 0 to 1. The panel summarizes lineage-defining marker patterns and supports the lineage-level annotation used throughout the study.

Abbreviations: CD, cluster of differentiation; HLA-DR, human leukocyte antigen-DR; NK, natural killer.

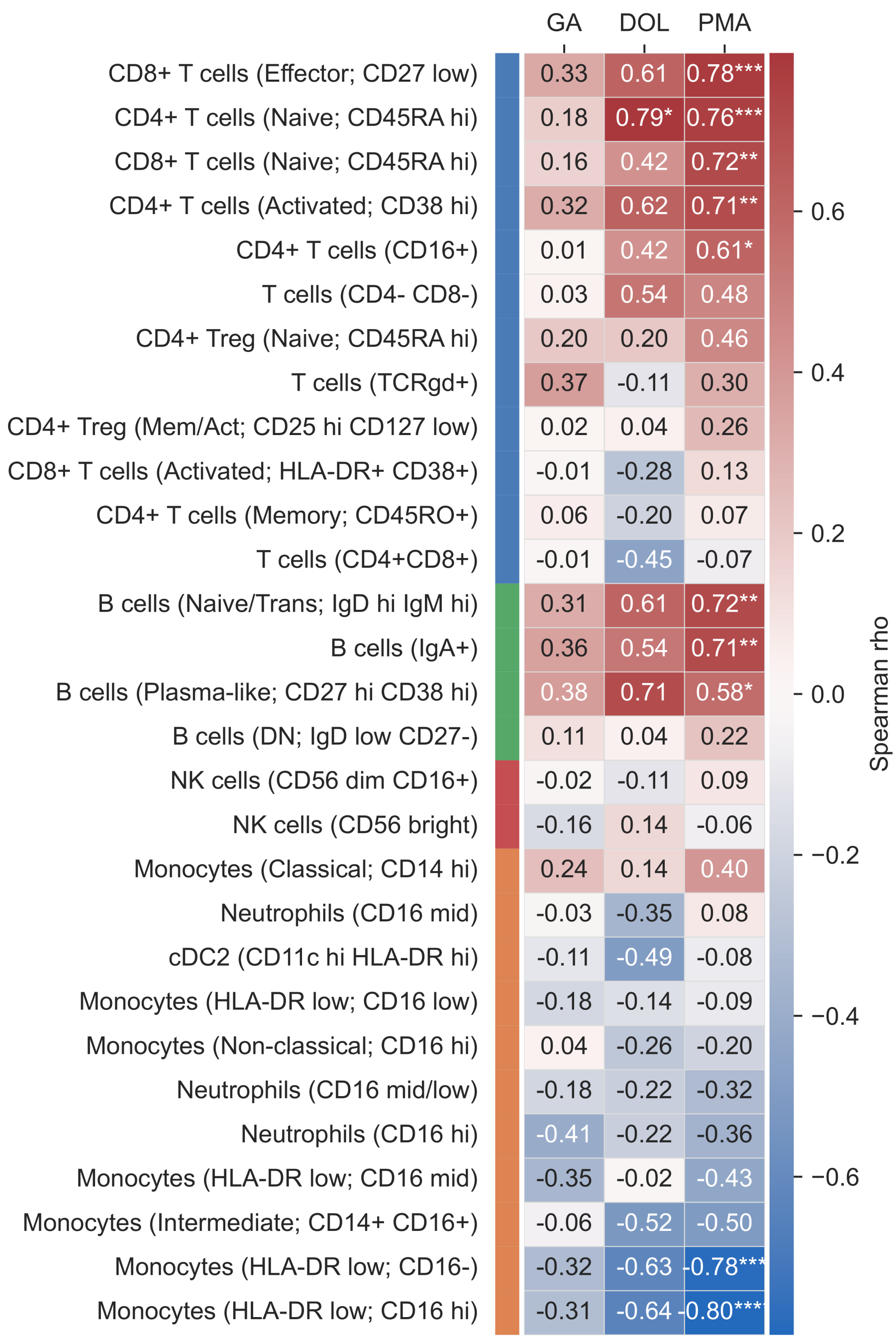

Figure S3. Developmental associations of immune cell subtypes with gestational age, day of life, and postmenstrual age.

Heatmap summarizing Spearman correlations between cell-subtype abundance and developmental time variables. Columns show gestational age (GA), day of life at sampling (DOL), and postmenstrual age at sampling (PMA). Rows show the final annotated cell subtypes ordered by lineage. Cell colors represent Spearman rho; overlaid values show the correlation coefficient, with asterisks indicating statistical significance after multiple-testing correction. The heatmap highlights coordinated postnatal maturation patterns, including increasing lymphoid features and decreasing HLA-DR-low monocyte/granulocyte-skewed features with developmental age.

Abbreviations: CD, cluster of differentiation; DOL, day of life; GA, gestational age; HLA-DR, human leukocyte antigen-DR; PMA, postmenstrual age.

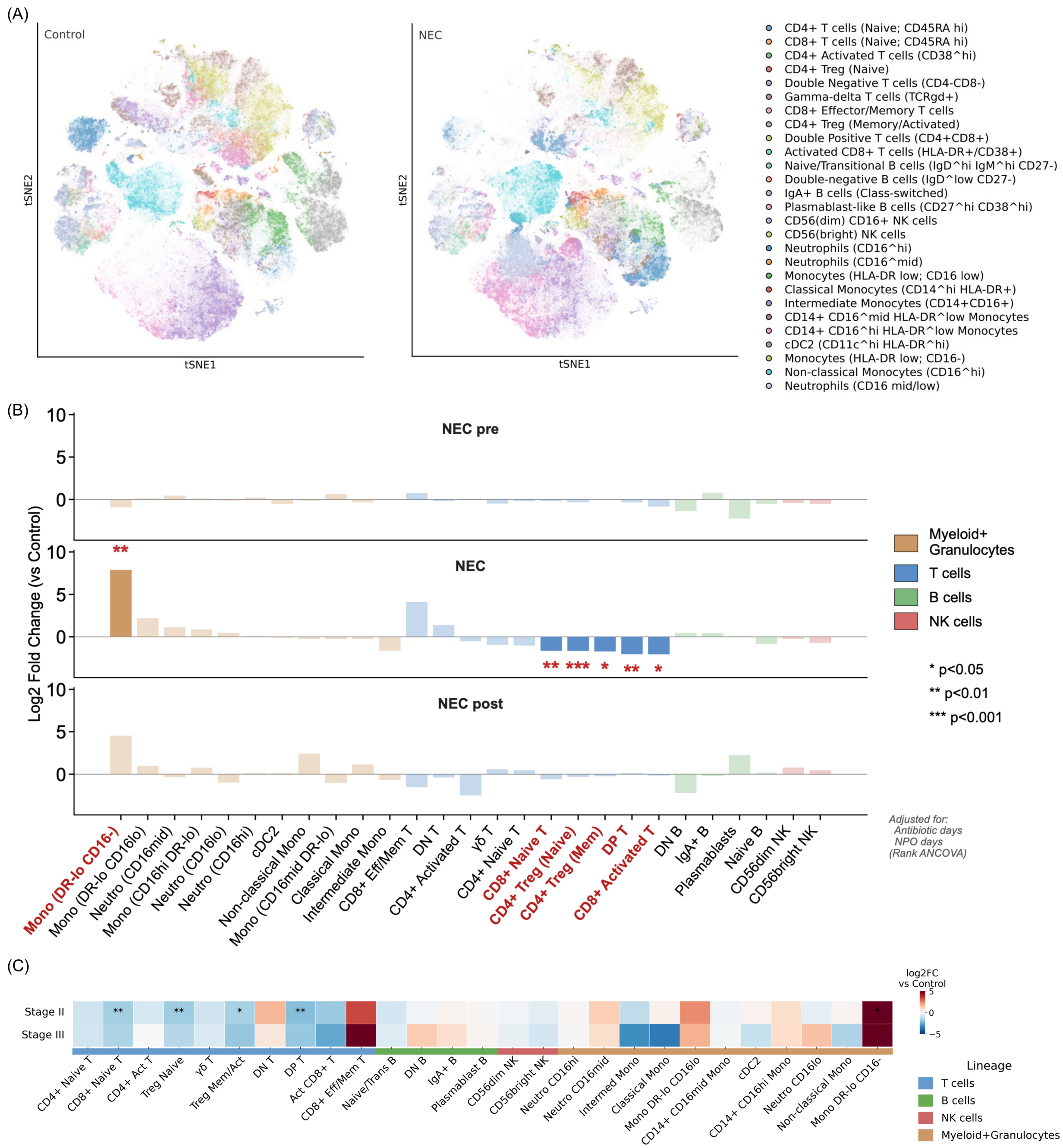

Figure S4. NEC-associated immune landscape and subtype-level disease patterns.

(A) Global tSNE projection comparing Control and NEC samples, with cells colored by the final 27 immune cell-subtype annotations. (B) Subtype-level log2 fold changes versus Control across NEC-pre, NEC, and NEC-post samples. Bars are colored by lineage, and asterisks denote statistically significant differences after multiple-testing correction. The analysis highlights a prominent expansion of HLA-DR-low CD16-negative monocytes at NEC onset and concurrent contraction of selected T-cell populations. (C) Heatmap of subtype abundance patterns across Control and NEC severity groups, showing the broader immune-state shift associated with NEC.

Abbreviations: CD, cluster of differentiation; HLA-DR, human leukocyte antigen-DR; NEC, necrotizing enterocolitis; NK, natural killer; tSNE, t-distributed stochastic neighbor embedding.

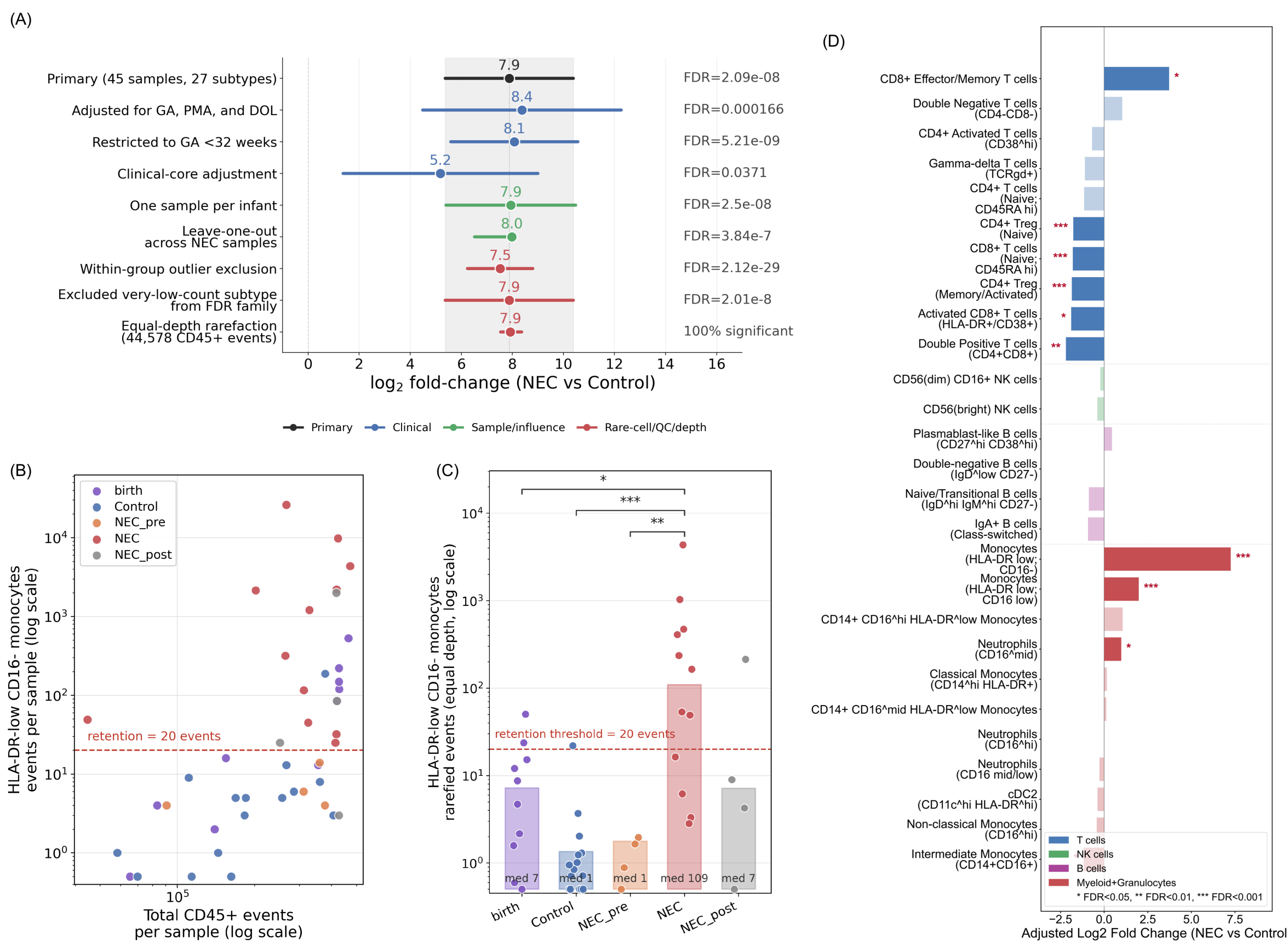

Figure S5. Robustness of the NEC-associated HLA-DR-low monocyte signal and clinical-core adjustment.

(A) Consolidated robustness forest plot for HLA-DR-low CD16-negative monocytes in NEC versus Control. The primary negative-binomial model and all sensitivity analyses yielded a positive and statistically significant effect, including one-sample-per-infant analysis, developmental covariate adjustment, restriction to gestational age <32 weeks, within-group outlier exclusion, clinical-core adjustment, leave-one-out analysis across NEC samples, exclusion of very-low-count subtypes from the FDR family, and equal-depth rarefaction. Points indicate log2 fold change and bars indicate 95% confidence intervals, observed leave-one-out range, or rarefaction range as indicated. (B) Relationship between HLA-DR-low CD16-negative monocyte event counts and total acquired CD45+ events per sample, demonstrating that the NEC enrichment is not explained by acquisition depth. (C) Equal-depth rarefaction analysis after down-sampling all samples to a common CD45+ event depth, confirming persistence of the NEC-associated enrichment. (D) Clinical-core adjusted differential-abundance analysis across all 27 subtypes using the final clinical covariate set.

Abbreviations: CD, cluster of differentiation; DOL, day of life; FDR, false discovery rate; GA, gestational age; HLA-DR, human leukocyte antigen-DR; NEC, necrotizing enterocolitis; PMA, postmenstrual age.

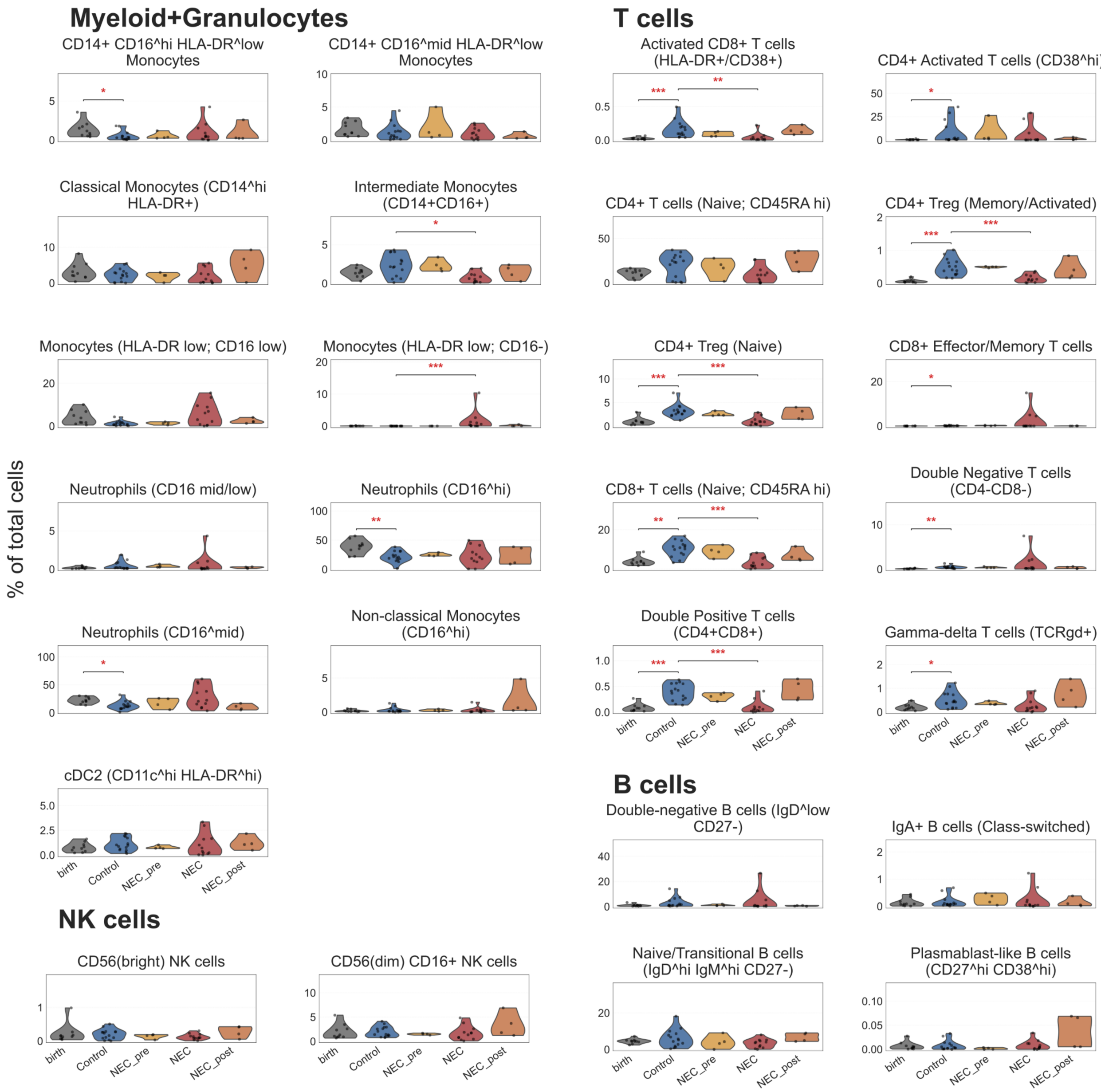

Figure S6. Full distribution of immune cell-subtype frequencies across sample groups. Violin and point plots showing the frequency distribution of each final annotated immune cell subtype across Birth, Control, NEC-pre, NEC, and NEC-post groups. Subtypes are grouped by lineage: Myeloid+Granulocytes, T cells, NK cells, and B cells. Individual points represent samples, and violins summarize the distribution within each group. Pairwise statistical annotations are shown for significant comparisons. This exhaustive panel provides distribution-level support for the subtype-level effect-size analyses.

Abbreviations: CD, cluster of differentiation; HLA-DR, human leukocyte antigen-DR; NEC, necrotizing enterocolitis; NK, natural killer.

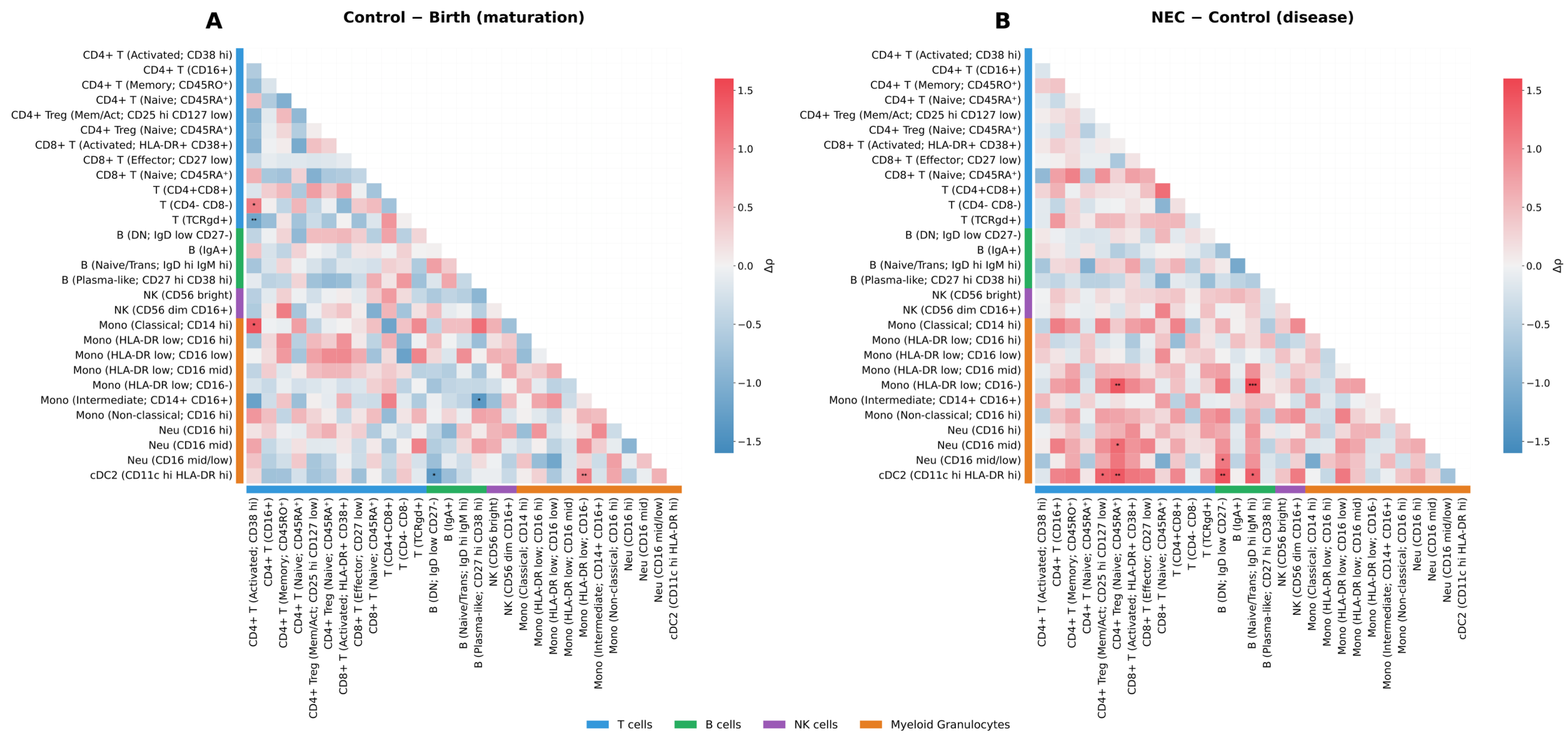

Figure S7. Developmental and NEC-associated rewiring of immune cell-correlation networks.

Heatmaps showing pairwise changes in immune cell-subtype correlations across developmental and disease contrasts. (A) Development-associated correlation changes, calculated as Control minus Birth. (B) NEC-associated correlation changes, calculated as NEC minus Control. Cell colors represent change in Spearman correlation (Δρ), with red indicating increased correlation and blue indicating decreased correlation. Asterisks denote edges with significant changes by Fisher r-to-z testing after false-discovery-rate correction. Colored bars indicate the lineage assignment of each cell subtype. These data support the conclusion that NEC remodels immune coordination in a pattern distinct from normal postnatal maturation.

All supplementary analyses used the 45 valid samples and OPTIMAL preprocessing unless otherwise stated. Group labels are Birth, Control, NEC-pre, NEC, and NEC-post.

Abbreviations: CD, cluster of differentiation; FDR, false discovery rate; HLA-DR, human leukocyte antigen-DR; NEC, necrotizing enterocolitis; Δρ, change in Spearman correlation coefficient.
